# Intraoperative Hyperspectral Imaging for Perfusion Assessment and Emerging Decision Support in Abdominal Surgery: A Systematic Review of Clinical Studies

**DOI:** 10.3390/diagnostics16091336

**Published:** 2026-04-29

**Authors:** Calin Muntean, Melania Veronica Ardelean, Vasile Gaborean, Alaviana Monique Faur, Catalin Vladut Ionut Feier

**Affiliations:** 1 Department III-Functional Sciences, Medical Informatics and Biostatistics, “Victor Babeş” University of Medicine and Pharmacy Timişoara, Eftimie Murgu Square No. 2, 300041 Timişoara, Romania; cmuntean@umft.ro; 2 Department V, Internal Medicine I-Discipline of Medical Semiology I, “Victor Babeş” University of Medicine and Pharmacy Timişoara, Eftimie Murgu Square No. 2, 300041 Timişoara, Romania; 3 Center of Advanced Research in Cardiology and Hemostasology, “Victor Babeş” University of Medicine and Pharmacy Timişoara, Eftimie Murgu Square No. 2, 300041 Timişoara, Romania; 4 Thoracic Surgery Research Center, “Victor Babeş” University of Medicine and Pharmacy Timişoara, Eftimie Murgu Square No. 2, 300041 Timişoara, Romania; 5 Department of Surgical Semiology, Faculty of Medicine, “Victor Babeş” University of Medicine and Pharmacy Timişoara, Eftimie Murgu Square No. 2, 300041 Timişoara, Romania; 6 Department of Doctoral Studies, “Victor Babeş” University of Medicine and Pharmacy Timişoara, Eftimie Murgu Square No. 2, 300041 Timişoara, Romania; alaviana.faur@umft.ro; 7 Abdominal Surgery and Phlebology Research Center, “Victor Babeş” University of Medicine and Pharmacy Timişoara, Eftimie Murgu Square No. 2, 300041 Timişoara, Romania; catalin.feier@umft.ro; 8 First Surgery Clinic, “Pius Brinzeu” Clinical Emergency Hospital, 300723 Timişoara, Romania

**Keywords:** hyperspectral imaging, abdominal surgery, colorectal surgery, perfusion assessment, liver transplantation, pancreatoduodenectomy, precision surgery, intraoperative imaging

## Abstract

**Background and Objectives:** Intraoperative assessment of tissue perfusion remains a decisive but imperfect step in abdominal surgery. Surgeons still rely heavily on visual judgement when choosing bowel transection lines, constructing anastomoses, judging intestinal viability, or assessing graft reperfusion, even though these decisions are directly linked to anastomotic leak, conduit ischemia, postoperative liver dysfunction, and graft failure. Hyperspectral imaging (HSI) is an emerging contrast-free optical technology that generates quantitative maps of tissue oxygenation, hemoglobin distribution, water content, and near-infrared perfusion. The present review was designed to evaluate whether clinical intraoperative HSI has matured sufficiently to support a focused systematic review topic in abdominal surgery and to synthesize the currently available human evidence. **Methods:** A literature search was conducted up to 20 February 2026 using combinations of the terms “hyperspectral imaging”, “HSI”, “abdominal surgery”, “colorectal”, “hepatectomy”, “transplantation”, “pancreatoduodenectomy”, “esophagectomy”, “mesenteric ischemia”, and “intraoperative”. Eligible records were original human clinical studies evaluating intraoperative HSI in abdominal or transplant-related operations with perfusion, oxygenation, or tissue viability as a central endpoint. Review articles, animal studies, non-surgical diagnostic studies, and single-patient case reports were excluded. Data were synthesized narratively because of major heterogeneity in indications, designs, devices, timing of measurements, and reported outcomes. **Results:** Thirteen studies published between 2019 and 2024 met the eligibility criteria, representing 391 patients. The literature covered colorectal resection, acute mesenteric ischemia, esophageal reconstruction with gastric or colonic conduits, pancreatoduodenectomy, pancreas transplantation, major hepatectomy, liver transplantation, and minimally invasive system validation. Across colorectal studies, HSI frequently demonstrated discordance between visually selected and objectively perfused transection lines, with clinically relevant strategy changes in a substantial proportion of patients. In ischemic and transplant settings, HSI discriminated poorly perfused tissue, identified low near-infrared perfusion values associated with early allograft dysfunction, and quantified reperfusion patterns after clamping or implantation. The evidence base was dominated by prospective single-center feasibility studies with small to moderate sample sizes, and no randomized trials were identified. **Conclusions:** Clinical intraoperative HSI in abdominal surgery is a genuinely niche yet rapidly expanding topic with a sufficient number of human studies to support a relevant systematic review. Current evidence consistently supports feasibility, quantitative perfusion discrimination, and plausible intraoperative utility, especially in colorectal and transplant-related surgery. However, the field remains methodologically heterogeneous, and the next research priority is multicenter standardization with clinically anchored thresholds and outcome-driven comparative studies.

## 1. Introduction

Abdominal surgery remains one of the clearest clinical environments in which real-time perfusion matters immediately and decisively. A bowel segment, gastric conduit, liver remnant, or transplanted graft may appear acceptable under white light and yet still harbor clinically relevant hypoperfusion that only becomes evident when anastomotic leakage, ischemic failure, or early graft dysfunction develops postoperatively [1,2]. Because of this, surgeons have long searched for adjunctive technologies that move intraoperative perfusion assessment beyond subjective visual inspection alone. Contemporary evidence from colorectal surgery shows that perfusion-guidance strategies can reduce anastomotic complications, especially when they support rather than merely confirm surgeon judgement, while the parallel literature on rectal surgery continues to reinforce the central role of microvascular adequacy in healing and leak prevention [3,4,5,6,7]. At the same time, acute mesenteric ischemia and hepatobiliary surgery remain settings in which delayed recognition of tissue compromise can be catastrophic, making objective perfusion mapping especially attractive [8,9,10,11]. These considerations explain why perfusion-imaging technologies have become one of the most active methodological frontiers in abdominal surgery and why niche, procedure-focused evidence syntheses are increasingly valuable for identifying where clinical translation is truly occurring [1,2,3].

Within this broader movement, hyperspectral imaging (HSI) has emerged as a particularly interesting platform because it can generate contrast-free, contact-free physiological maps from tissue reflectance across multiple wavelengths. Unlike methods that depend on dye injection, repeated acquisitions are possible without cumulative contrast exposure, and multiple parameters can be derived from a single image set, including superficial oxygen saturation, near-infrared perfusion-related indices, tissue hemoglobin, and water content [1,2]. For surgeons, this matters because perfusion is rarely a purely binary phenomenon; rather, it is a spatially heterogeneous continuum that can change after devascularization, clamping, reperfusion, or conduit creation. Other intraoperative imaging adjuncts such as ultrasound and laser speckle technologies also provide clinically useful perfusion information, but they answer somewhat different questions and have distinct workflow constraints [8,12,13,14,15,16,17]. HSI therefore occupies a complementary niche within the intraoperative imaging ecosystem, offering a surface-based, physiology-oriented map that is intuitive to interpret visually while still being amenable to quantitative analysis and, increasingly, computational augmentation [1,2,13,18,19,20,21,22,23].

From a biophysical perspective, the most frequently reported abdominal HSI variables should be interpreted as superficial optical biomarkers rather than direct measurements of full-thickness organ perfusion [24,25,26,27]. StO2 is derived primarily from the wavelength-dependent absorption behavior of oxyhemoglobin and deoxyhemoglobin; the near-infrared perfusion index (NIR-PI) reflects tissue reflectance in the near-infrared range and is usually interpreted as a blood-volume/perfusion-related signal; the organ hemoglobin index (OHI) reflects total hemoglobin content; and the tissue water index (TWI) is influenced by water absorption, particularly around the longer near-infrared wavelengths. These variables are clinically attractive because they can be displayed as intuitive maps, but their interpretation must consider that visible/NIR light interrogates predominantly superficial tissue layers and is affected by scattering, surface contamination, and acquisition geometry [28,29].

Nevertheless, adoption of any new intraoperative technology in abdominal surgery depends not only on technical elegance but also on whether it addresses an unresolved clinical need better than established alternatives. Indocyanine green fluorescence angiography (ICG-FA) has already become the most familiar comparator in colorectal and rectal surgery because it provides dynamic visualization of vascular inflow and has accumulated a large body of observational and randomized evidence [3,4,5,6,7,14,15]. Systematic reviews and meta-analyses now suggest that ICG-FA can reduce anastomotic leakage in selected settings, particularly left-sided and rectal resections, although technique standardization and interpretation remain imperfect [4,5,6]. This is precisely where HSI becomes relevant as a distinct hot topic rather than a redundant one. It offers dye-free repeatability, provides multi-parametric tissue characterization instead of a single fluorescence signal, and may be better suited to objective threshold development and machine learning integration [1,2]. For a review topic to be meaningful, however, it must sit in the narrow space between being too broad to synthesize coherently and too immature to support more than anecdotal conclusions. The abdominal HSI perfusion literature now appears to occupy that space, which is why it warrants targeted appraisal rather than casual mention within broader imaging overviews [1,2,3].

A second reason this topic is timely is that abdominal surgery presents several very different but mechanistically related use-cases for perfusion imaging. In colorectal surgery, the central issue is safe transection-line and anastomotic-site selection. In acute mesenteric ischemia, the challenge is differentiating salvageable from non-salvageable bowel rapidly enough to guide resection and second-look planning. In hepatopancreatobiliary surgery, surgeons may need to understand the physiological effect of vascular clamping, arterial inflow compromise, or parenchymal redistribution, whereas transplantation introduces the additional complexity of ischemia–reperfusion injury and early graft function prediction [9,10,11,12,13]. These are not interchangeable scenarios, but they all depend on the same fundamental premise: surface physiology may reveal clinically important information before gross macroscopic deterioration becomes obvious. That shared premise creates a coherent conceptual umbrella for systematic review while still preserving a clinically meaningful niche. It also explains why broader surgical HSI reviews are informative but insufficient for a reader specifically interested in abdominal perfusion-guided decision-making [1,2].

The currently available published literature further suggests that HSI is no longer limited to engineering demonstrations or ex vivo tissue classification. Broader reviews of surgical HSI have documented rapid growth in experimental and translational work, while adjacent abdominal imaging studies show that surgeons increasingly expect intraoperative technologies to be quantitative, reproducible, and decision-relevant rather than merely illustrative [1,2,3,8,14]. Yet an important gap remains between broad technological enthusiasm and practical topic selection for a publishable systematic review. A topic can feel novel while still lacking enough mature clinical studies to sustain a serious evidence synthesis; conversely, a topic can have enough studies but no longer be sufficiently distinctive if it has already been repeatedly reviewed at the same level of specificity. In the present context, broader HSI reviews and mixed-modality perfusion syntheses do exist, but they do not replace a dedicated examination of clinical intraoperative HSI for abdominal perfusion-guided decision-making across procedures in which perfusion itself is the operative question [1,2,3]. That distinction is important for both scientific framing and manuscript originality.

Recent computational HSI work further strengthens the rationale for this translational framing. Lai et al. summarized the movement of HSI and machine learning pipelines toward clinical tissue diagnostics [30], while Huang et al. demonstrated a software-based approach for transforming white-light gastrointestinal endoscopy images into hyperspectral-like information for improved disease detection [31]. Foundational upper-GI endoscopic work by Wu et al. also showed that hyperspectral endoscopic imaging can support early identification of esophageal squamous neoplasia [32]. Although these studies are not intraoperative abdominal perfusion studies, they contextualize the broader gastrointestinal trajectory of HSI: from hardware-centered image acquisition toward computationally enhanced, quantitative tissue diagnostics.

Accordingly, the aim of the present manuscript was twofold. First, we sought to evaluate whether intraoperative HSI for perfusion-guided abdominal surgical decision-making still represents a defensible niche systematic review topic in the current literature landscape. Second, we aimed to synthesize the available clinical evidence in a structured way that would be useful for manuscript development, topic refinement, and future protocol planning. Rather than treating all surgical HSI uses as interchangeable, we focused specifically on human intraoperative abdominal studies in which perfusion, oxygenation, reperfusion, or tissue viability formed a meaningful clinical target [1,2,3,15].

## 2. Materials and Methods

### 2.1. Review Design and Conceptual Framework

This manuscript was developed as a systematic review of clinical studies. The guiding concept was that hyperspectral imaging should be evaluated according to the specific intraoperative decisions it is meant to support in abdominal surgery: selection of bowel transection margins, assessment of intestinal viability, perfusion optimization of gastric or colonic conduits, appraisal of liver or pancreas graft reperfusion, and detection of perfusion compromise after vascular clamping or implantation. A topic defined in this way is methodologically stronger than a generic review of “all surgical HSI”, because it narrows the clinical context while still preserving enough heterogeneity to assess the breadth of human application. The review therefore targeted abdominal and transplant-related procedures in which perfusion, oxygenation, or tissue viability were central operative concerns. This scope allowed inclusion of colorectal surgery, upper gastrointestinal reconstruction when based on abdominal conduits, hepatobiliary surgery, pancreatoduodenectomy, acute intestinal ischemia surgery, pancreas transplantation, and liver transplantation. The review was not designed as a diagnostic accuracy study, a hardware engineering review, or a scoping review of preclinical platforms. Instead, it sought to answer a pragmatic clinical question: has intraoperative HSI generated enough human evidence to justify a dedicated niche systematic review topic in abdominal surgery, and what do those studies collectively show? A systematic review structure was chosen because the field is emerging, the studies are dispersed across procedure-specific journals, and the novelty claim must rest on a transparent identification of the available human literature rather than on anecdotal impressions. The conceptual framework also shaped the later synthesis, which focused not only on feasibility and image acquisition, but also on the degree to which HSI produced quantitative discrimination, prompted a change in operative strategy, or correlated with postoperative outcomes.

### 2.2. Information Source and Search Strategy

The literature search was conducted in PubMed, Scopus, and Web of Science, which were selected to capture both biomedical and surgical-engineering publications relevant to intraoperative HSI. Embase and the Cochrane Library were not used as primary search platforms because the question focused on original clinical intraoperative HSI studies rather than intervention trials or diagnostic-accuracy meta-analysis; nevertheless, reference-list screening and related-article searches were performed to reduce the chance of omitting relevant reports. The final search was performed on 20 February 2026, covering records from database inception to that date. Search strings combined “hyperspectral imaging” OR “HSI” with abdominal and intraoperative terms including “colorectal surgery”, “mesenteric ischemia”, “esophagectomy”, “gastric conduit”, “colon interposition”, “pancreatoduodenectomy”, “pancreas transplantation”, “hepatectomy”, “liver transplantation”, “abdominal surgery”, “surgical”, and “intraoperative”. Duplicate records were removed before screening. The strategy followed the PRISMA 2020 reporting framework and was designed to capture human intraoperative studies while excluding preclinical engineering, pathology-only, and non-surgical endoscopic literature outside the scope of this review. The review protocol was registered on the Open Science Framework with the identifier https://osf.io/9at7d (accessed on 8 April 2026). The PRISMA checklist is provided in Table S1.

### 2.3. Eligibility Criteria

Eligibility criteria were defined before full-text synthesis so that the final review would remain clinically coherent and methodologically focused. Studies were included when they met all of the following conditions: they were original human clinical studies; they evaluated hyperspectral imaging intraoperatively during an abdominal, upper gastrointestinal conduit, hepatopancreatobiliary, intestinal ischemia, or abdominal transplant-related operation; they reported perfusion, oxygenation, reperfusion, tissue viability, or a directly related intraoperative decision target; and they were indexed in PubMed, Scopus, and Web of Science with sufficient bibliographic detail to support accurate reference construction. Both prospective and retrospective clinical studies were eligible, as were pilot studies, feasibility cohorts, technical validation studies embedded in human operations, and small consecutive case series, provided that HSI was actually used during the operation and patient-level clinical context was present. The decision to include feasibility and pilot studies was deliberate, because the field remains young and excluding them would erase much of the currently available abdominal evidence. However, single-patient case reports were excluded to prevent the review from being driven by anecdotal rather than cumulative data.

Animal experiments, ex vivo laboratory analyses without an intraoperative human component, purely engineering papers without patient application, pathology-only tissue classification studies, non-surgical diagnostic endoscopy studies, conference abstracts without full bibliographic maturity, narrative reviews, systematic reviews, editorials, commentaries, and letters were also excluded. Studies focused on non-abdominal specialties, such as thyroid, skin, neurosurgery, or cardiovascular monitoring, were not considered, even if they used HSI, because they were outside the intended surgical domain. When transplantation studies contained both bench and recipient measurements, they were retained only if intraoperative recipient or implantation-stage assessment formed part of the clinical workflow, since these studies remained relevant to perfusion-guided abdominal decision-making. No rigid minimum sample size threshold was imposed, because the developmental stage of the field would have made such a filter arbitrary and potentially misleading. Instead, relevance was judged on the basis of clinical applicability, intraoperative integration, and availability of numerical outcome information. Because single-patient case reports may contain early clinical signals in niche technologies, their exclusion is acknowledged as a trade-off: it reduced anecdotal weighting but may have omitted rare early observations on unexpected intraoperative utility or outcome signals.

### 2.4. Study Selection and Data Extraction

**Figure 1:**
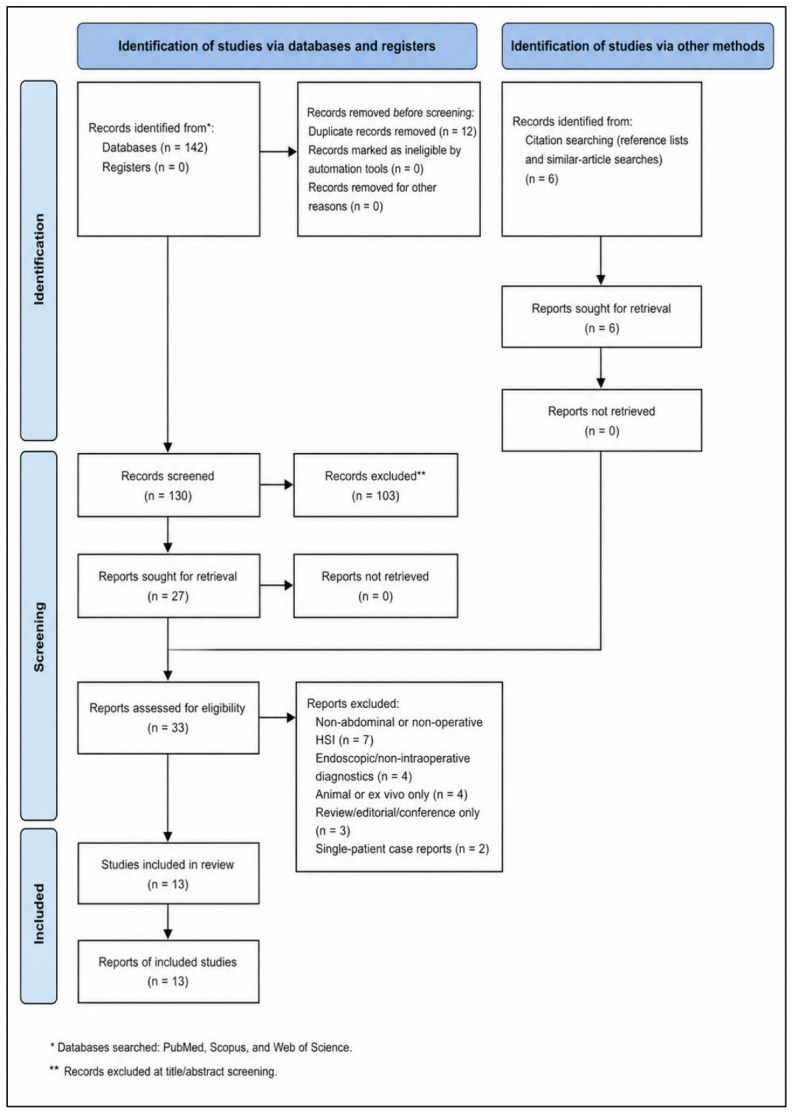
PRISMA 2020 flow diagram for study selection.

Study selection followed a staged manual process appropriate for a focused systematic review. First, titles and abstracts retrieved through the PubMed, Scopus, and Web of Science strategy were screened for obvious relevance to abdominal surgery and intraoperative his (Figure 1). Records centered on non-human work, pathology bench analysis, basic science instrumentation, or non-abdominal indications were removed at this level. Second, potentially eligible articles underwent full-text review through the linked PubMed records, publisher pages, PubMed Central, Scopus, and Web of Science entries when available. Full-text review was especially important because many HSI publications use feasibility-oriented titles that do not immediately reveal whether the article contains true intraoperative human data, clinically relevant outcomes, or only technical system testing. During full-text assessment, each article was mapped against the predefined inclusion criteria and assigned to one of three categories: include, exclude, or background-only reference. Included studies were then entered into a structured extraction grid developed for this manuscript. The extraction fields were chosen to support a clinically meaningful narrative synthesis and included publication year, lead author, surgical indication, operative setting, study design, cohort size, type of HSI platform, operative approach, timing of image acquisition, HSI parameters reported, comparator or decision target, principal numerical findings, intraoperative impact, and postoperative or graft-related outcomes. Additional notes were captured when studies contained distinctive workflow features, such as serial clamping measurements, comparison with an established open system, or analysis during machine perfusion and recipient reperfusion. Because the present review was intended both as a manuscript and as a model for topic development, special attention was paid to variables that help a future author understand whether the field is sufficiently mature: how often HSI altered decisions, how often it produced interpretable maps, whether threshold-like behavior was reported, and whether any downstream clinical outcome signal was observed. Data extraction emphasized factual reporting from the published articles and did not infer unreported values. When outcomes were heterogeneously presented, the original metric style was preserved in the extraction phase to avoid introducing false comparability across studies that used different endpoints, units, or temporal windows.

### 2.5. Quality Appraisal and Strategy for Data Synthesis

Given the heterogeneity of procedures, endpoints, and study designs, the synthesis strategy remained intentionally descriptive rather than meta-analytic. In addition to narrative comparison, methodological quality was appraised using the Methodological Index for Non-Randomized Studies (MINORS), a validated tool for non-randomized surgical research [33]. For each included study, the eight non-comparative MINORS domains were considered: clearly stated aim, inclusion of consecutive patients, prospective data collection, endpoints appropriate to the aim, unbiased assessment of endpoints, adequate follow-up, loss to follow-up below 5%, and prospective sample size calculation. Because none of the included studies was a randomized comparative trial, the comparative MINORS domains were not used for scoring but were considered narratively when interpreting evidence strength. This formal appraisal was added to make study-level limitations more transparent and to prevent feasibility findings from being interpreted as definitive effectiveness evidence.

## 3. Results

### 3.1. Overview of the Evidence Base

The literature search identified 13 eligible human clinical studies published between 2019 and 2024, together representing 391 patients [16,17,18,19,20,21,22,23,24,25,26,27,34]. The evidence base was concentrated in Europe and was dominated by single-center prospective feasibility cohorts, pilot studies, or consecutive observational series. Colorectal surgery was the most developed clinical area, but the final set of studies also encompassed acute mesenteric ischemia, gastric and colonic conduit evaluation after esophageal reconstruction, pancreatoduodenectomy, pancreas transplantation, major hepatectomy, liver transplantation, and validation of minimally invasive HSI platforms. No randomized controlled trials were identified. Across the included literature, the most consistently reported HSI outputs were superficial tissue oxygen saturation (StO2) and near-infrared perfusion indices, often supplemented by organ or tissue hemoglobin and tissue water parameters (Table 1).

**Figure 2:**
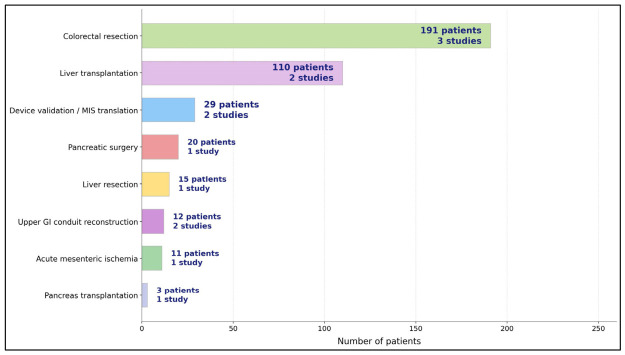
Evidence by domain of HSI abdominal surgery.

Table 1 shows that the clinical HSI literature in abdominal surgery is no longer confined to isolated anecdotes. Even after exclusion of single-patient case reports, 13 human studies and 391 patients were identified, which is enough to support a meaningful systematic review question rather than a purely illustrative narrative. The pattern of publication is also informative. The earliest studies were strongly feasibility-oriented and were concentrated in colorectal resection and intestinal ischemia, where the clinical relevance of perfusion mapping is immediately intuitive. Over time, the field expanded into technically demanding settings such as pancreatoduodenectomy, pancreas transplantation, major hepatectomy, and liver transplantation, indicating that the technology is being tested not only in routine bowel surgery but also in operations where perfusion failure carries particularly severe consequences. Table 1 also reveals the developmental stage of the evidence base. Most studies were prospective, observational, and single-center, with cohort sizes ranging from very small exploratory series to a larger colorectal cohort of 115 patients and a transplant cohort of 73 recipients (Figure 2).

### 3.2. Intraoperative Workflow and HSI Acquisition Patterns

Table 2 emphasizes that the most important unifying feature across studies is not the operation itself but the workflow logic of HSI use. Regardless of whether investigators studied colorectal transection lines, ischemic bowel, graft reperfusion, or conduit selection, HSI was consistently deployed at a moment when the surgeon needed to judge whether tissue perfusion was adequate enough to proceed, revise, or extend the operative plan. This table also shows that most groups relied on a similar physiological vocabulary. Tissue oxygen saturation and near-infrared perfusion were the dominant outputs, usually complemented by hemoglobin- and water-related indices when the platform allowed. The recurrence of these parameters across colorectal, pancreatic, hepatic, and transplant applications suggests that HSI is evolving toward a shared intraoperative language rather than fragmented, procedure-specific readouts. Another important insight is timing. Many studies purposely measured tissue after a physiological stress maneuver: marginal artery division, gastroduodenal artery clamping, vessel clamping during conduit preparation, or reperfusion after transplantation.

**Figure 3:**
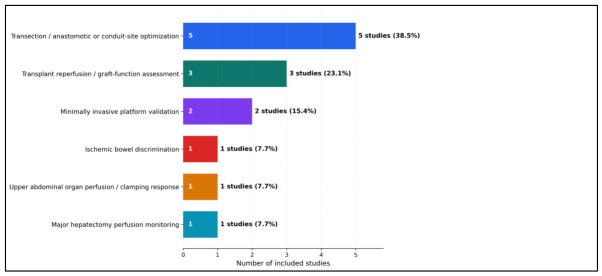
Quantitative findings and clinical impact of intraoperative hyperspectral imaging across abdominal surgery studies.

A further source of heterogeneity is platform dependence. Most included clinical applications used the TIVITA system or closely related derivatives, while fewer studies evaluated alternative acquisition frameworks or minimally invasive adaptations such as EX-MACHYNA or HSI-MIS. This dominance matters because spectral range, calibration procedure, illumination geometry, camera-to-tissue distance, acquisition time, and proprietary parameter reconstruction can all influence StO2, NIR-PI, OHI, and TWI values. Therefore, thresholds derived from one platform should not be assumed to transfer directly to another device without cross-platform validation, phantom calibration, and standardized acquisition protocols (Figure 3).

### 3.3. Quantitative Findings and Clinical Translation

Table 3 provides the strongest argument that HSI is more than a feasibility technology. Across several studies, the system did not merely produce interpretable maps; it revealed quantitative discrepancies with conventional intraoperative assessment that were large enough to influence management. In colorectal surgery, mismatches between clinically chosen and HSI-defined transection lines were common, and one large series showed that nearly half of cases did not have full agreement between subjective and objective perfusion assessment. In acute mesenteric ischemia, HSI discriminated poorly perfused from viable bowel using significant differences in tissue saturation and near-infrared perfusion. In pancreatoduodenectomy, it identified clinically important flow compromise during gastroduodenal artery clamping and supported median arcuate ligament release in selected patients. In conduit surgery, HSI enabled proximal revision of the anastomosis site in colon interposition and quantified marked oxygenation gradients in gastric conduits. In hepatic and transplant applications, low near-infrared values were not only measurable but also prognostically meaningful, correlating with biochemical injury, early allograft dysfunction, or worse overall survival. Collectively, these findings indicate that HSI already has a credible translational footprint.

### 3.4. Risk of Bias Assessment

MINORS appraisal confirmed that the current literature is methodologically useful for feasibility and signal detection but remains limited for causal inference. Most studies had clearly stated aims and clinically relevant endpoints, but recurrent limitations included single-center recruitment, small cohorts, absent prospective sample size calculations, limited blinding of outcome assessment, and heterogeneous follow-up. The highest-scoring evidence came from larger prospective observational colorectal and transplant cohorts, whereas very small feasibility series were most vulnerable to selection bias and imprecision (Table 4).

## 4. Discussion

The principal finding of this review is that intraoperative HSI has moved beyond isolated technical curiosity and now represents a coherent abdominal surgical evidence cluster with clinically relevant use-cases [16,17,18,19,20,21,22,23,24,25,26,27,34]. Across colorectal, ischemic, hepatopancreatobiliary, conduit, and transplant settings, the common value proposition was not simply prettier imaging, but the ability to identify perfusion heterogeneity that could plausibly influence operative judgement. This is important because existing perfusion assessment literature has already established that subjective visual assessment alone is imperfect and that quantitative or semi-quantitative adjuncts can improve decision-making in bowel surgery [3,4,5,6,7,15]. HSI appears to contribute to this field by adding a dye-free, repeatable, multi-parametric map rather than a single fluorescence snapshot. At the same time, the review also clarifies that the current evidence base remains predominantly exploratory. Most studies were small, single-center, and non-randomized, and many were designed to demonstrate physiologic discrimination or workflow feasibility rather than definitive outcome reduction. The appropriate conclusion, therefore, is neither overenthusiastic adoption nor dismissal, but recognition that abdominal HSI has now reached the stage where targeted multicenter validation is both possible and justified [1,2,3,16,17,18,19,20,21,22,23,24,25,26,27,34].

Colorectal surgery remains the strongest current clinical anchor for HSI, and the colorectal studies in this review help explain why [16,20,22]. Anastomotic leakage is a complication with direct consequences for morbidity, reintervention, cost, and oncologic recovery, and it has therefore become the proving ground for intraoperative perfusion technologies [3,4,5,6,7,15]. Several included HSI studies showed that the surgeon’s intended transection line did not always match the perfusion border demonstrated by hyperspectral assessment, sometimes leading to proximal adjustment or to explicit recognition of marginally perfused bowel [16,20,22]. This pattern mirrors the broader ICG-FA literature, where the most reproducible value of perfusion imaging has often been based on changing the operative plan rather than providing simple visual reassurance [4,5,6,7,14,15]. The distinctive contribution of HSI is that it may support this decision without dye administration and with the added possibility of physiologic quantification. That feature could become increasingly important if future trials attempt to standardize perfusion thresholds, compare technologies directly, or integrate optical data into predictive algorithms rather than relying solely on surgeon interpretation of grayscale or fluorescence intensity changes [1,2].

The hepatopancreatobiliary and transplant studies broaden the importance of HSI beyond bowel anastomosis research and suggest that the modality may be particularly valuable where ischemia–reperfusion phenomena are spatially complex and clinically consequential [18,19,23,26,27]. In pancreatoduodenectomy, HSI was used to interrogate organ perfusion after clamping maneuvers and to reveal clinically important celiac-axis compromise [18]. In major hepatectomy, the technology was linked to perfusion and oxygenation mapping during vascular manipulations [23], while in pancreas and liver transplantation it was evaluated as a marker of reperfusion quality and early graft function [19,26,27]. These applications align conceptually with other abdominal imaging approaches, including liver microcirculation assessment and intraoperative ultrasound, but HSI offers the added advantage of simultaneous oxygenation- and perfusion-related surface mapping without contact or contrast injection [8,12,13]. Importantly, however, these domains also highlight a current limitation: clinical endpoints remain heterogeneous, and the distance between optical parameter change and patient-important outcome is still shorter in theory than in proven causal evidence. Future studies will need more standardized endpoints if HSI is to move from promising adjunct to evidence-based routine tool [1,2,3].

The field is also clearly moving toward technological convergence with minimally invasive surgery and computational analysis [21,24]. Laparoscopic validation studies showed that HSI can be translated from open systems into minimally invasive environments, although calibration differences and parameter agreement still need refinement [21,24]. This matters because any abdominal imaging technology that cannot migrate into laparoscopic and robotic workflows will struggle to achieve broad future relevance. More broadly, the current literature suggests that the most promising future direction is not merely image acquisition but standardized interpretation. HSI generates rich optical data, yet the clinical literature still uses variable thresholds, heterogeneous acquisition timings, and inconsistent reporting of how measurements altered operative choice. Broader surgical HSI reviews have reached a similar conclusion, emphasizing the need for workflow standardization, prospective validation, and integration with analytics or artificial intelligence [1,2]. Thus, the next phase of this field will likely depend less on proving that HSI can visualize perfusion and more on demonstrating when, how, and by how much HSI-guided actions improve outcomes compared with existing perfusion tools or expert clinical judgement alone [1,2,3,4,5,6,21,24,25,26,27].

### 4.1. Decision-Support Versus Observational Use of HSI

A clearer distinction is required between HSI as an observational physiologic mapping tool and HSI as a true intraoperative decision-support tool. In the present evidence base, documented operative strategy changes were concentrated in a small subset of studies, most notably Barberio et al. in colorectal surgery, Moulla et al. during pancreatoduodenectomy with suspected celiac-axis compromise, and Zimmermann et al. during colon interposition. In contrast, several studies used HSI primarily for feasibility, device validation, physiologic mapping, or postoperative association testing without a predefined HSI-triggered operative algorithm. The conclusions of this review were therefore moderated to state that HSI shows emerging decision-support potential rather than established broad clinical effectiveness.

### 4.2. Comparison with Competing Intraoperative Optical Modalities

HSI should be considered one member of a broader intraoperative optical-imaging ecosystem rather than a universal replacement for other tools. ICG fluorescence angiography provides dynamic angiographic inflow information but requires exogenous dye and is usually interpreted semi-quantitatively. Laser speckle contrast imaging can provide high-temporal-resolution surface microcirculation maps, whereas diffuse reflectance spectroscopy offers point-based or limited-field estimates of tissue oxygenation. Fluorescence imaging with targeted or metabolic probes can support oncologic margin visualization, and optical coherence tomography offers high-resolution structural information at limited penetration depth. The main advantage of HSI is repeatable, dye-free, wide-field, multi-parameter surface physiology; its main limitation is that visible/NIR HSI remains depth-limited and sensitive to surface and motion artefacts [28,29,35,36,37,38], as seen in Table 5.

### 4.3. Technical Artefacts, ROI Variability, and Reproducibility

Several technical factors can confound intraoperative HSI interpretation. Respiratory motion, peristalsis, cardiac pulsation, and instrument movement may cause spatial blurring and spectral mixing, particularly in systems requiring sequential acquisition. Surface blood, irrigation fluid, bile, smoke, or serosal glare can alter reflectance spectra and distort parameter estimates. Illumination inhomogeneity and camera-to-tissue angle are especially important in laparoscopic fields, where light delivery is directional and the working distance is constrained. ROI selection is another major source of heterogeneity: some studies used fixed anatomic landmarks, others manually selected visually relevant regions, and others used software-guided demarcation. Future protocols should therefore prespecify ROI geometry, distance, acquisition timing after vascular ligation or reperfusion, artefact exclusion rules, and whether HSI values are interpreted as absolute thresholds, relative gradients, or changes from baseline.

### 4.4. Priorities for a Multicenter Colorectal HSI Trial

Based on the colorectal studies synthesized here, an upcoming multicenter randomized trial should prioritize a limited number of reproducible HSI triggers rather than an unrestricted multi-parameter display. Candidate triggers include an HSI-defined perfusion border that differs from the surgeon-selected transection line by more than 5 mm, low StO2 at the proposed anastomotic site, and low NIR-PI relative to adjacent well-perfused bowel. Because absolute cutoffs remain platform-specific and insufficiently validated, the first trial should use a prespecified HSI-guided algorithm based on local perfusion gradients and border displacement, with device-specific exploratory thresholds recorded for later validation. The recommended primary endpoint is 30-day clinically relevant anastomotic leak after colorectal resection. Secondary endpoints should include change in the transection line, reoperation, diverting stoma, surgical site infection, readmission, time added to operation, interobserver agreement, and health-economic outcomes.

This review has several limitations that should be acknowledged. First, the included evidence was heterogeneous in procedure type, timing of measurements, HSI platforms, reported parameters, ROI definitions, and outcome definitions, which precluded formal meta-analysis and limits direct cross-study comparability. Second, most reports were small single-center observational studies focused on feasibility or early translational performance rather than hard comparative endpoints. Third, although the search was expanded to PubMed, Scopus, and Web of Science, Embase and the Cochrane Library were not primary search platforms, so database-related retrieval bias cannot be fully excluded. Fourth, single-patient case reports were excluded; this choice improved cumulative evidentiary rigor but may have omitted early clinical signals in rare abdominal HSI applications. Fifth, the dominance of TIVITA-related platforms and several geographically concentrated research groups increases the risk of publication, confirmation, and center-expertise bias. Finally, HSI parameters are surface-weighted optical biomarkers and may not represent deeper mural, parenchymal, or graft perfusion in all clinical contexts.

## 5. Conclusions

Intraoperative hyperspectral imaging for perfusion assessment and emerging decision support in abdominal surgery remains a timely and clinically relevant systematic review topic because the abdominal perfusion literature now comprises a sufficiently distinct cluster of human studies. The available evidence supports feasibility, repeated detection of visually occult perfusion differences, and early decision-support signals in selected colorectal, pancreatoduodenectomy, conduit, and transplant scenarios. However, most studies remain observational, single-center, and platform-concentrated, and HSI should not yet be presented as a broadly proven outcome-improving technology. The most meaningful next step is a rigorously designed multicenter colorectal trial with standardized acquisition, prespecified HSI action thresholds, transparent ROI rules, and patient-centered endpoints such as clinically relevant anastomotic leak.

## Figures and Tables

**Table 1:** Characteristics of the included clinical studies.

Study	Procedure/Domain	Design	Sample	Setting	Primary Clinical Focus
Jansen-Winkeln 2019 [16]	Colorectal resection	Open-label, single-arm, non-randomized intervention trial	24	Germany, single center	HSI-based determination of bowel transection margin after marginal artery division
Mehdorn 2020 [17]	Exploratory laparotomy for acute mesenteric ischemia	Prospective observational pilot study	11	Germany, single center	Discrimination of viable versus poorly perfused/necrotic intestine
Schwandner 2021 [34]	Esophagectomy with gastric conduit	Feasibility case series	4	Germany, single center	Assessment of gastric sleeve oxygenation before esophagogastric anastomosis
Moulla 2021 [18]	Pancreatoduodenectomy	Prospective pilot study	20	Germany, single center	Perfusion of upper abdominal organs during GDA clamping and celiac stenosis detection
Sucher 2022 [19]	Simultaneous pancreas-kidney transplantation	Prospective pilot series	3	Germany/Austria collaboration, single center	Pancreas graft reperfusion and intestinal anastomosis monitoring
Jansen-Winkeln 2022 [20]	Colorectal resection	Prospective observational study	115	Germany, single center	Border-line definition and agreement with clinically chosen resection margin
Pfahl 2022 [21]	Gastrointestinal cancer resections (resectate validation)	Clinical evaluation study	10	Germany, single center	Validation of a handheld laparoscopic HSI camera against an approved open system
Barberio 2022 [22]	Elective colorectal surgery	Prospective observational study	52	Italy/Switzerland network, single center clinical cohort	Quantification of bowel perfusion and clinical-versus-hyperspectral transection-line discordance
Felli 2022 [23]	Major hepatectomy for malignant liver lesions	Prospective preliminary clinical trial	15	Multicenter European collaboration	Prediction of short-term postoperative liver dysfunction after major hepatectomy
Thomaßen 2023 [24]	Gastrointestinal resections	Prospective in vivo validation study	19	Germany, single center	In vivo comparison of minimally invasive HSI (HSI-MIS) with open HSI
Zimmermann 2023 [25]	Colon interposition after esophagectomy	Retrospective/observational clinical series	8	Germany, single center	Selection of optimal colonic conduit segment and anastomotic site
Vogt 2024 [26]	Human liver transplantation	Prospective observational study	37	Germany, single center	Prediction of early allograft dysfunction across bench, NMP, and recipient reperfusion phases
Lederer 2024 [27]	Orthotopic liver transplantation	Observational cohort study	73	Germany/Austria collaboration	Prediction of early allograft dysfunction and overall survival after recipient reperfusion

**Table 2:** Intraoperative HSI workflow, acquisition strategy, and decision targets.

Study	Platform/Device	Operative Context	Timing of HSI Acquisition	Reported HSI Outputs	Decision Target/Comparator
Jansen-Winkeln 2019 [16]	Open HSI camera (500–1000 nm)	Open colorectal surgery	Every minute for 15 min after vessel division	StO2/perfusion maps from false-color imaging	Compared surgeon-planned line versus HSI border line
Mehdorn 2020 [17]	TIVITA-type open HSI system	Open ischemia surgery	Intraoperative assessment during laparotomy	Tissue saturation, NIR perfusion index, OHI, TWI, reflectance spectra	Compared HSI values across macroscopic perfusion groups
Schwandner 2021 [34]	TIVITA Tissue HSI camera	Thoraco-abdominal upper GI reconstruction	Before gastroesophageal anastomosis; 4 ROIs per sleeve	StO2 and NIR perfusion index	Compared oral versus aboral regions around clinically determined line
Moulla 2021 [18]	TIVITA Tissue System	Open pancreatic surgery	Before and after GDA clamping, including serial measurements up to 30 min	StO2 and OHI, with perfusion-oriented organ mapping	Assessed effect of clamping and need for MAL division in celiac artery stenosis
Sucher 2022 [19]	TIVITA HSI system	Transplant implantation	15 min after reperfusion	StO2, NIR, OHI, TWI	Compared graft pancreas and graft duodenum with recipient jejunum at the anastomosis
Jansen-Winkeln 2022 [20]	Compact HSI camera	Open colorectal surgery before anastomosis	Immediately after vessel division; border assessed from 1 to 3 min	Quantitative tissue oxygenation/perfusion border	Compared clinical transection line with HSI demarcation
Pfahl 2022 [21]	Laparoscopic HSI camera vs. clinically approved open HSI	Minimally invasive system validation using GI resectates	Immediate intraoperative/resectate comparison	StO2, NIR-PI, OHI, TWI, spectral RMSE	Technical comparison rather than therapeutic decision change
Barberio 2022 [22]	TIVITA system	Elective colorectal surgery for neoplasia or diverticular disease	Before anastomosis creation	StO2 heat map and hyperspectral transection line	Resection performed at HSI line when discrepancy > 5 mm
Felli 2022 [23]	Intraoperative HSI (EX-MACHYNA framework)	Open liver resection	Intraoperative hepatic assessment around resection/reperfusion phases	TWI, OHI, StO2, NIR	Correlated HSI with postoperative biochemical and clinical endpoints
Thomaßen 2023 [24]	HSI-MIS vs. HSI-Open	Intraoperative minimally invasive validation	ROI matched intraoperatively between systems	StO2, NIR-PI, TWI, OHI, spectral RMSE; original and PLS-calculated values	Technical equivalence assessment
Zimmermann 2023 [25]	Intraoperative HSI	Complex reconstructive upper GI surgery	After clamping middle colic vessels; root and tip analyzed	Perfusion maps of colon conduit root and tip	Potential change in conduit segment or anastomosis site
Vogt 2024 [26]	HSI acquisition software for graft parenchyma	Liver graft assessment during preservation and implantation	Bench preparation, during NMP when applicable, and after recipient reperfusion	StO2, THI, NIR, TWI	Compared parameters by preservation strategy and EAD status
Lederer 2024 [27]	Intraoperative HSI after reperfusion	Recipient implantation phase	15 min after donor-liver reperfusion	Perfusion-related HSI variables with focus on NIR	Related low versus high NIR to postoperative graft function

**Table 3:** Main quantitative findings, operative impact, and reported clinical outcomes.

Study	Main Quantitative Finding	Intraoperative Impact	Postoperative/Clinical Outcome	Interpretive Note
Jansen-Winkeln 2019 [16]	Perfusion margin visualized directly in 20/24; software-assisted in 4/24. Median line deviation 1 mm (range −13 to 13). Perfusion dropped up to 12% within first 10 mm distal to border.	Showed objective mismatch between visual and HSI-based resection planning in all cases.	Feasibility study; postoperative outcome not primary endpoint.	Established early proof that visible macroscopic judgement may differ from perfusion imaging.
Mehdorn 2020 [17]	Tissue saturation 0.70 vs. 0.45 (*p* = 0.002) and NIR perfusion index 0.58 vs. 0.23 (*p* < 0.001) in viable versus poorly perfused segments; 630-nm spectral peak increased in necrotic tissue.	Supported objective discrimination between salvageable and non-salvageable bowel.	Suggests value for bowel preservation and resection planning in AMI.	One of the first human ischemia-focused HSI abdominal studies.
Schwandner 2021 [34]	Median StO2 69% aboral vs. 53% oral to the line; median NIR perfusion 80 vs. 82. No anastomotic failure in 4/4 patients.	Provided immediate spatial oxygenation profile of the conduit.	No leaks observed, but sample size too small for outcome inference.	Illustrates upper GI conduit application of HSI.
Moulla 2021 [18]	4/20 had celiac artery stenosis; 2 showed liver oxygenation decrease after clamping. In one patient StO2 improved from 61% to 73% after MAL division.	HSI exposed clinically meaningful clamping-related hypoperfusion not evident from routine inspection alone.	Potentially useful adjunct when arterial inflow adequacy is uncertain during PD.	Important niche HPB application linked to operative decision change.
Sucher 2022 [19]	Pancreas graft StO2 92.6% ± 10.45. Anastomotic StO2 67.46% ± 5.60 in graft duodenum vs. 75.93% ± 4.71 in jejunum (*p* < 0.001); TWI 0.63 ± 0.09 vs. 0.72 ± 0.09 (*p* < 0.001).	Showed reproducible graft and anastomotic perfusion mapping immediately after implantation.	All 3 had uneventful postoperative course; 1 reversible Banff 1a rejection.	Demonstrates transplant-facing extension of HSI beyond resection surgery.
Jansen-Winkeln 2022 [20]	Clear line in 105/115; plateau after 3 min. Clinical line matched HSI in 58/105 (55.2%), lay fully within less-perfused area in 23/105 (21.9%), and was crossed irregularly in 24/105 (22.8%).	Showed frequent mismatch between visual planning and objective perfusion.	Authors argued HSI could help prevent postoperative complications, especially leak.	Largest colorectal HSI cohort in the review.
Pfahl 2022 [21]	MAE: StO2 11% ± 7, NIR-PI 14 ± 3, OHI 14 ± 5, TWI 10 ± 2; RMSE 0.10 ± 0.03 (500–750 nm) and 0.15 ± 0.06 (750–1000 nm).	Supported feasibility of laparoscopic translation of HSI hardware.	No direct postoperative endpoint; key value was platform validation.	Relevant because future abdominal HSI adoption depends on MIS compatibility.
Barberio 2022 [22]	Anastomotic leak in 1 patient. CTL/HTL discrepancy in 26/52; mean StO2 54.55% ± 21.30 with discrepancy vs. 65.10% ± 21.30 without (*p* < 0.001). Neoadjuvant radiochemotherapy group had StO2 51.41% ± 23.41 vs. 60.51% ± 24.98 (*p* = 0.010).	Demonstrated direct operative consequences when HSI and visual assessment diverged.	Low leak count, but study designed primarily for perfusion characterization.	One of the clearest decision-guidance colorectal reports.
Felli 2022 [23]	NIR higher in unhealthy tissue (*p* = 0.003). StO2 negatively correlated with postoperative ALT (r = −0.602); ΔStO2 positively correlated with ALP (r = 0.594). TWI correlated with reintervention; OHI with sepsis and liver failure.	Linked optical signatures to clinically important postoperative events.	Suggests HSI may serve as an intraoperative hepatic risk-stratification tool.	Key bridge from feasibility to outcome-associated liver surgery data.
Thomaßen 2023 [24]	Original MAE: StO2 17.2% ± 7.7, NIR-PI 16.0 ± 9.5, TWI 18.1 ± 7.9, OHI 14.4 ± 4.5. PLS-improved MAE: 12.6% ± 5.2, 10.3 ± 6.0, 10.6 ± 5.1, 11.6 ± 3.0; RMSE 0.14 ± 0.06.	Showed that MIS-adapted HSI can approximate open-platform outputs with algorithmic optimization.	Facilitates future real-time laparoscopic adoption.	Technical maturation study with direct relevance to clinical scalability.
Zimmermann 2023 [25]	Anastomotic leak in 1/8 (12.5%); no conduit necrosis. Anastomosis site changed proximally in 2/8. No intraoperative need to change conduit side.	Provided actionable perfusion information during an uncommon but high-risk reconstruction.	No patient required conduit removal, diversion, or stent placement.	Useful example of HSI in niche reconstructive abdominal surgery.
Vogt 2024 [26]	NMP n = 31, SCS n = 6. EAD 36.7% in NMP vs. 50% in SCS (*p* = 0.6582). NIR lower in EAD at 1 h (18.6 vs. 28.3, *p* = 0.0468), 2 h (19.4 vs. 37.1, *p* = 0.0011), 4 h (26.0 vs. 40.3, *p* = 0.0080), and after reperfusion (13.0 vs. 30.6, *p* = 0.0212).	Suggested objective perfusion thresholds may predict graft dysfunction.	Supports HSI as a peri-implantation graft assessment adjunct.	One of the strongest transplant datasets in the field.
Lederer 2024 [27]	Expanded-criteria donors 41/73 (56.9%); mean MELD 22 ± 10; EAD 18/73 (25%). Low NIR after reperfusion associated with EAD and lower overall survival (*p* = 0.049).	Extended transplant evidence from feasibility to prognostic relevance.	Recipients with low NIR showed more pronounced reperfusion injury in labs and worse survival.	Currently the largest purely intraoperative transplant cohort identified.

**Table 4:** Risk of Bias Assessment.

Study	Design Type	Consecutive/Prospective Reporting	Endpoint Assessment	Follow-Up/Outcome Completeness	MINORS Score *	Main Risk-of-Bias Concern
Jansen-Winkeln 2019 [16]	Prospective non-comparative	Partial/yes	Unblinded imaging-supported endpoint	Limited clinical outcome focus	11/16	Feasibility design; no outcome-powered control group
Mehdorn 2020 [17]	Prospective pilot	Likely/yes	Macroscopic viability groups, unblinded	Short clinical follow-up	11/16	Small acute ischemia cohort; no independent blinded endpoint adjudication
Schwandner 2021 [34]	Feasibility case series	Unclear/yes	ROI-based oxygenation profile	Complete but n = 4	8/16	Very small sample; no comparative decision pathway
Moulla 2021 [18]	Prospective pilot	Likely/yes	Clamping-related physiologic endpoint	Postoperative outcomes described	12/16	Selected HPB population; non-randomized decision impact
Sucher 2022 [19]	Prospective pilot series	Unclear/yes	Graft and anastomotic perfusion mapping	Complete but n = 3	8/16	Very small transplant series; descriptive only
Jansen-Winkeln 2022 [20]	Prospective observational	Likely/yes	HSI border vs. clinical line	Outcome linkage limited	12/16	Large cohort but no randomized management algorithm
Pfahl 2022 [21]	Clinical validation	Yes/yes	Device agreement metrics	Outcome not primary	11/16	Technical validation rather than clinical effectiveness
Barberio 2022 [22]	Prospective observational	Yes/yes	Clinical vs. HSI transection line	Postoperative leaks reported	13/16	Decision algorithm present but no blinded comparison group
Felli 2022 [23]	Prospective preliminary trial	Likely/yes	Biochemical/postoperative associations	Short-term outcomes reported	11/16	Small heterogeneous liver-resection cohort
Thomaßen 2023 [24]	Prospective in vivo validation	Yes/yes	Open vs. MIS parameter agreement	Outcome not primary	11/16	Technical equivalence study; not outcome-guided
Zimmermann 2023 [25]	Observational clinical series	Unclear/retrospective component	Conduit perfusion and anastomosis site	Clinical outcomes described	9/16	Small uncommon reconstruction cohort; selection bias likely
Vogt 2024 [26]	Prospective observational	Likely/yes	EAD association across phases	Early graft function reported	12/16	Preservation-strategy heterogeneity; non-randomized
Lederer 2024 [27]	Observational cohort	Likely/partly prospective	EAD and survival association	Clinically meaningful endpoints	13/16	Largest transplant cohort but still single-network observational evidence

* MINORS was applied using the eight non-comparative domains (maximum score 16). Comparative domains were considered narratively when relevant because none of the included studies was a randomized comparative trial.

**Table 5:** Comparison with Competing Intraoperative Optical Modalities.

Modality	Typical Signal	Approximate Depth/Field Behavior	Strengths	Key Workflow Limitations
HSI	Reflectance-derived StO2, NIR-PI, OHI, TWI	Surface-weighted; visible/NIR light mainly samples superficial millimeters	Dye-free, repeatable, multi-parametric, wide-field mapping	Acquisition time, motion sensitivity, surface fluids, calibration and platform dependence
ICG fluorescence angiography	Exogenous dye fluorescence	Dynamic vascular inflow; depth depends on NIR fluorescence and tissue optical properties	Familiar colorectal workflow; strong clinical comparator literature	Requires dye, timing/dose variability, usually semi-quantitative
Laser speckle contrast imaging	Speckle decorrelation from moving red cells	Very superficial microvascular flow mapping with high temporal resolution	Real-time flow-sensitive imaging	Sensitive to motion and illumination; limited adoption in abdominal surgery
Diffuse reflectance spectroscopy	Spectral absorption/scattering signatures	Point-based or limited-field oxygenation assessment	Quantitative tissue oxygenation potential	Limited spatial mapping unless integrated into imaging systems
Targeted/PpIX fluorescence	Probe-specific fluorescence	Probe- and wavelength-dependent tumor or tissue contrast	Oncologic margin and molecular guidance potential	Requires probe, regulatory pathway, and timing standardization
OCT	Backscattered light microstructure	High spatial resolution but shallow penetration	Margin and layer-structure assessment	Small field of view; structural rather than perfusion-focused

## Data Availability

No new data were created or analyzed in this study.

## Supplementary Materials

The following supporting information can be downloaded at: https://www.mdpi.com/article/10.3390/diagnostics16091336/s1, Table S1: PRISMA 2020 Checklist.
